# Malleination and Tuberculination in Point of a Judicial Examination

**Published:** 1896-07

**Authors:** 


MALLEINATION AND TUBERCULINATION IN POINT OF A JUDICIAL EXAMINATION. The practice of malleination and of tuberculination from a judicial point of view is not without importance.

It may be advantageous to consider the two preceding successively:

*Malleination.* Will an expert who can discover no clinical symptom whatsoever of glanders in the animal examined be justified in concluding the existence of this affection upon the single fact of reaction from mallein? I think not, notwithstanding the positive opinion of Nocard and Roux as to the value of malleination as a means of diagnosis in glanders. In a judicial examination, which has for its aim the establishment of the existence or absence of a defect which prevents the sale of an animal, there is no room for a middle term; the defect exists or it does not exist. In spite of the strongest presumption that glanders is present when the mallein affirms it, the competent administrative authorities in Belgium, as well as in France, in Germany, in Switzerland, and elsewhere, do not consider animals affected with glanders which show no exterior visible symptom of the disease, although they have reacted sufficiently after mallein. These animals are designated as suspected of being affected, and under this head are subjected to suitable sanitary police regulations. The reservation of the authorities has its ground in the lack of success established by certain investigators—Foth, Müller, and others—as well as in the opinion of most of the Russian veterinarians, who are competent to judge, that mallein, such as is in the market at present, has not given results uniformly free from objection. This reservation is all the more justified, as it in no way interferes with the action of the sanitary police, and it agrees perfectly with the proposition adopted by the congress in Berne, in 1895, that "mallein is a powerful means of establishing a diagnosis of the disease in cases of *suspected* glanders"—that is, in the cases where there is some outward visible clinical manifestation. But what ought the expert to conclude in the case supposed? He will simply make a verbal report, reserve his decision until a second examination, and make use of the pound if that has not been already done. In the meantime some one or other external symptom will probably manifest itself, and the expert will then be able to make a certain decision—that is, to declare that the disease is certainly present. He will be enabled to temporize, as he will not have to consider the expense resulting from idleness or the slaughter of the animal, since, having reacted from mallein, it will fall under the application of Article 319 of the Penal Code and of Articles 3 and 60 of the Royal Decision of

September 20, 1883, by virtue of which it must be quarantined for sixty days after malleination, if no external symptom of glanders appears during that period. After this quarantine has ended with a negative result, the expert will be legally justified in concluding that the disease is absent. On the contrary—in case some clinical symptom appears during the period of quarantine—the animal will be killed by order of the authorities, according to Article 8 of the Royal Decision before mentioned. The autopsy will furnish the investigator opportunity to complete his diagnosis.

*Tuberculination.* Here, as in the case of glanders, a judicial decision is to be given which will be justified by the same considerations as those which were explained in reference to glanders. The same question arises in this place: Can an expert who has obtained a sufficient reaction after tuberculin, in an animal, conscientiously deduce from it that the animal is affected with this disease? Assuredly so, if at the same time some visible symptom of a chronic disease of the lungs is noted. But not, if to the characteristic reaction which tuberculin reveals there is present absolutely no clinical manifestation whatsoever of any deep-seated affection of the lung. Why these contradictory answers, when, in the cases mentioned, tuberculosis will be sufficiently established in accordance with the regulation of October 30, 1895, by virtue of which every animal is considered as affected with tuberculosis which, on the tuberculin-test, presents the characteristic reaction? It makes little difference whether there are visible symptoms coexisting with the reaction, the latter is plainly present and the disease is proved. This statement cannot be refuted if one restricts himself to the definition of the sanitary police. Article 2 of the regulation mentioned above does not permit any equivocation on the subject. Nevertheless it is necessary to remember that the sanitary police make no distinction as to the seat of the malady, whether it be confined to the thorax exclusively or localized in the abdomen; whether it be peritoneal or intestinal makes no difference. The affection exists, the sanitary police simply apply the the rule without distinction. But while the sanitary police do not take account of the situation of the disease, it is quite a different matter in regard to a *redhibitory* defect. According to the law relating to tuberculosis, phthisis can only be followed by redhibition if the seat of the disease is upon the pectoral organs of the respiratory apparatus. It makes no difference whether it has invaded other organs or not. Every doubt ought to be dissipated as to this precise point; tuberculosis, although present in other organs, is only redhibitory when it attacks the intrathoracic organs. It is vain to pretend that abdominal tuberculosis being much more frequently unobserved than tuberculosis of the lungs and pleura, ought at least, under the same head as the last, to be subject to redhibition. The range of a royal decree accepted as a law cannot be extended under the pretext that it does not embrace sufficiently all the cases which it has for its object. If the law is inadequate, it is the business of the government to extend it; and until that time it must be executed in the letter and in the spirit which was given to it when it was enacted. Tuberculination discloses tuberculosis wherever its seat; it may at the time of the judicial investigation be confined to the abdominal cavity, although this seldom happens, while the respiratory organs remain untouched. The investigator would not then be justified in concluding that the defect existed on the basis of a single reaction producing a rise in temperature of at least 1.4° above the normal. What is the obligation of the expert then in a similar case—that is to say, when the presence of tuberculosis is confirmed by tuberculin reaction without the seat of the disease being determined? In view of the buyer who demands it or of the judge who has commissioned him, the expert should have the animal in question put into quarantine and reserve final decision, as in the case of glanders which had alone been detected by malleination. As in the case of glanders, there need be no fear here of the losses resulting from the sequestration of the animal, for it must be quarantined as soon as it has reacted, in conformity to Article 18 of the law of October 30, 1895, concerning tuberculosis. In the meantime some clinical manifestation revealing lesions in the chest will probably appear, and an absolute decision affirming the existence of the defect can be legally made. In view of the authorities the judicial expert will have to acquit himself of the obligation imposed by Article 10 of the aforementioned law; he must then notify the official inspector of the district, who will make further disposition of the matter.—*Annales de Méd. Vétérinaire*, Jan. and Feb. 1896.

